# Cognitive impairment in multiple sclerosis: An exploratory analysis of environmental and lifestyle risk factors

**DOI:** 10.1371/journal.pone.0222929

**Published:** 2019-10-21

**Authors:** Maria Pia Amato, Elio Prestipino, Angelo Bellinvia, Claudia Niccolai, Lorenzo Razzolini, Luisa Pastò, Roberto Fratangelo, Laura Tudisco, Mattia Fonderico, Paolo Luca Mattiolo, Benedetta Goretti, Giovanni Bosco Zimatore, Nunzia Alessandra Losignore, Emilio Portaccio, Francesco Lolli

**Affiliations:** 1 Department NEUROFARBA, Section of Neurosciences, University of Florence, Florence, Italy; 2 IRCCS Fondazione Don Carlo Gnocchi, Florence, Italy; 3 SOD Neurological Rehabilitation, Careggi University Hospital, Florence, Italy; 4 Operative Unit of Neurology, Dimiccoli General Hospital, Barletta, Italy; 5 SOC Neurology, San Giovanni di Dio Hospital, Florence, Italy; Heinrich-Heine-Universitat Dusseldorf, GERMANY

## Abstract

**Background:**

Many potentially modifiable risk factors for MS are investigated. It is not known, however, if these factors also apply to MS-related cognitive impairment (CI), a frequent consequence of MS.

**Objective:**

The aim of our study was to assess risk factors for CI in MS patients, focusing on environmental exposures, lifestyle and comorbidities.

**Methods:**

We included MS patients referring to MS Centers in Florence and Barletta between 2014 and 2017. Neuropsychological performance was assessed through the Rao’s battery and Stroop test, cognitive reserve (premorbid intelligence quotient–IQ) was evaluated using the National Adult Reading Test (NART). Potential risk factors were investigated through a semi-structured questionnaire.

**Results:**

150 patients were included. CI was detected in 45 (30%) subjects and was associated with older age (p<0.005), older age at MS onset (p = 0.016), higher EDSS score (p<0.005), progressive disease course (p = 0.048) and lower premorbid IQ score (p<0.005). As for risk factors, CI was related with lower physical activity in childhood-adolescence (p<0.005). In women, hormonal therapy resulted to be protective against CI (p = 0.041). However, in the multivariable analysis, the only significant predictors of CI were older age (p<0.05; OR 1.06, 95% CI 1.02–1.10) and lower premorbid IQ (p<0.05; OR 0.93, 95% CI: 0.88–0.98). Removing IQ from the model, CI was associated with higher EDSS (p = 0.030; OR 1.25, 95% CI 1.02–1.53) and, marginally, previous physical activity (p = 0.066; OR 0.49, 95% CI: 0.23–1.05)

**Conclusions:**

Our findings suggest that physical activity in childhood-adolescence could be a contributor to cognitive reserve building, thus representing a potential protective factors for MS-related CI susceptible to preventive strategies.

## Introduction

Multiple sclerosis (MS) is a neuroinflammatory and neurodegenerative demyelinating disease of the central nervous system (CNS) with onset usually in young adulthood with a female to male ratio of nearly three to one, especially in relapsing-remitting MS (RRMS) patients [1].

While the pathogenesis of the disease is most likely autoimmune, the etiology is multifactorial: multiple factors, both genetic and environmental, determine disease risk and interact with one another in a complex manner. Beyond genetic susceptibility, many environmental, potentially modifiable factors have been identified to play a role in of the development MS, and, in a few cases, also in the prognosis of the disease. The most widely investigated include Epstein-Barr virus (EBV) infection, smoking, low levels of vitamin D, obesity and comorbidities [2].

Cognitive impairment (CI) is a common feature of MS, affecting approximately 40% to 70% of patients at any time in their disease course [3]. The neuropsychological pattern is usually characterized by deficits in information processing speed and complex attention, episodic memory, executive functions and visuospatial abilities. Regardless of the disease duration and level of physical disability, CI has a significant functional impact and negatively affects various aspects of the patients' quality of life and lifestyle. Compared with cognitively preserved patients, patients with CI experience in fact a lower level of activity and participation in daily life, work and social activities [4]. Moreover, CI interferes with coping strategies, adherence to treatments and capability to benefit from rehabilitative strategies [5,6]. A few studies tried to focus on potential risk factors or protective factors for MS-related CI [3], even if without strong results. In this respect, especially information on environmental and lifestyle risk factors would be of help in adopting preventive strategies, identifying subjects at higher risk for CI and fostering assessment and management strategies.

The objective of this exploratory study is to identify possible risk factors/protective factors for CI in a clinical cohort of MS patients, focusing on potentially modifiable, environmental and lifestyle factors.

## Patients and methods

### Subjects

We included MS patients with relapsing–remitting (RR), primary and secondary progressive MS (PPMS, SPMS) [7] referred to the local MS center in the period from 2014 to 2017. Inclusion criteria were: diagnosis of MS (2010 revisions of the McDonald criteria), age ≥ 18 years, interval between diagnosis and inclusion into the study ≤ 10 years, no relapses or steroid treatment in the month before the neuropsychological testing, no history of developmental intellectual disability, complicated brain trauma, psychosis and dementing disease other than MS. All the patients underwent neuropsychological testing and answered a detailed interview on hypothesized risk factors at the presence of a caregiver. The study was approved by the Ethics Committee of the University of Florence, and written informed consent was obtained from the patients and their caregivers.

### Clinical and neuropsychological assessment

Demographic and clinical data were prospectively collected every six months and in occasion of relapses and stored in an electronic database [8]. Information including disease onset, disease course, treatments, relapses and disability level assessed on the Expanded Disability Status Scale (EDSS) [9] was reviewed by the neurologist of the center. A well-trained psychologist administered the patients the Brief Repeatable Battery of Neuropsychological Tests (BRB) [10] and Stroop test [11].

The BRB assesses the cognitive domains most frequently impaired in MS and incorporates tests of verbal memory (Selective Reminding Test [SRT]); visual memory (10/36 Spatial Recall Test [SPART]); complex attention and information processing speed (Paced Auditory Serial Addition Test [PASAT]; Symbol Digit Modalities Test [SDMT]); and verbal fluency (Word List Generation). The Stroop Color and Word Test (SCWT) [11] assesses complex attention and aspects of executive functioning such as the ability to inhibit cognitive interference. Failure of a test was defined when the score was below the 5^th^ or above the 95^th^ percentile (1.65 SD), as appropriate, employing normative Italian values [12]. Patients were classified as having CI when they failed at least three neuropsychological tests, based on previous studies where using the same neuropsychological battery we found that less than 5% of healthy controls failed more than three tests [13–15].

Cognitive reserve was evaluated by the National Adult Reading TEST (NART) [16] and the Cognitive leisure activity questionnaire [17].

Depression was assessed through the Montgomery-Åsberg Depression Rating Scale (MADRS) [18] and fatigue was self-assessed by the patients through the Fatigue Severity Scale (FSS) [19].

The neuropsychological test battery was administered in a single session. Breaks were provided upon the subject’s request or when fatigue was evident.

A semi-structured interview, developed *ad hoc* for the study, was administered by the psychologist to each patient in the presence of the caregiver, to investigate previous and current exposure to hypothesized risk factors. These included cardiovascular risk factors and comorbidities, psychiatric disorders, history of brain trauma, hormonal therapies, body mass index (BMI), diet and lifestyle (type of diet, vitamin D supplementation due to low vitamin levels, caffeine intake, smoking, alcohol consumption, cannabis and substance abuse, leisure activities, current and childhood-adolescence physical activity), as well as family history of MS, psychiatric disorders and dementia. Physical activity was assessed on patient report based on the International Physical Activity Questionnaire (IPAQ) [20] developed as an instrument for cross-national monitoring of physical activity and inactivity. Results were reported as categories: 3. high activity levels; 2. moderate activity levels 1. low activity levels. Category 3 on IPAQ means vigorous intensity activity on at least 3 days achieving a minimum total physical activity of at least 1500 metabolic equivalent of task (MET) minutes a week or 7 or more days of any combination of walking, moderate intensity or vigorous intensity activities, achieving a minimum total physical activity of at least 3000 MET minutes a week. Category 2 is defined as three or more days of vigorous intensity activity and/or walking of at least 30 minutes per day or five or more days of moderate intensity activity and/or walking of at least 30 minutes per day or five or more days of any combination of walking, moderate intensity or vigorous intensity activities achieving a minimum total physical activity of at least 600 MET minutes a week. Finally, category 1 of physical activity is represented by patients’ activities that do not meet any of the abovementioned criteria.

The whole assessment required about 2 hours with an average of 1.15 hours for the test battery, fatigue and depression assessment, and 45 minutes for the interview.

The interview used in this study is available as supplementary material.

### Statistical analysis

Demographic and clinical characteristics were described as frequency (percentage) and mean ± standard deviation (SD). Group comparisons were assessed through the Pearson’s chi^2^ Student t and Mann–Whitney U tests when appropriate.

Possible predictors of CI were assessed through a stepwise multivariable logistic model, including the presence of CI as dependent variable and all the variables that were significant in the univariate analysis as covariates. Results are expressed as odds ratios (ORs) or estimated means, and 95% confidence intervals (CIs). P-values less than 0.05 were considered significant.

## Results

All the patients accepted the study procedures. One hundred and fifty MS patients were included; 45 (30%) were classified as cognitively impaired and 105 (70%) as cognitively preserved. The main demographic and clinical characteristics of the study sample are depicted on Table 1. Compared with cognitively preserved patients, patients with CI were older (p = 0.003), had a higher age at MS onset (p = 0.016), higher EDSS score (p = 0.001), progressive disease course (p = 0.048) and a lower premorbid IQ score (p = 0.004).

**Table 1:** Demographic and clinical characteristics of the study sample.

		Total Sample (n = 150)	Cognitively impaired (#45, 30%)	Cognitively preserved (#105, 70%)	*p*
**Age, years (mean ± SD)**		44.9 ± 11.1	48.9 ± 12.4	43.1 ± 10.1	0.003
**Sex (M/F)**		47/103	11/34	36/69	0.234
**Education, years (mean ± SD)**		12.6 ± 3.8	11.8 ± 4.3	12.9 ± 3.5	0.101
**Disease duration, years (mean ± SD)**		11.2 ± 9.3	11.7 ± 9.8	11.0 ± 9.2	0.690
**Age at onset (mean ± SD)**		33.6 ± 10.4	37.0 ± 10.9	32.2 ± 9.8	0.016
**EDSS median (IQR)**		2.0 (1.5–4.0)	3.0 (2.0–5.5)	2.0 (1.0–3.1)	0.001
**Disease course # (%)**		RR132 (88%) CP 18 (12%)	RR 36 (80%) CP 9 (20%)	RR 96 (91.4%), CP n = 9 (8.6%)	0.048
**FSS (mean ± SD)**		4.5 ± 1.9	4.9 ± 1.7	4.3 ± 2.0	0.089
**Moderate/Severe Depression # (%)**		11 (7.3%)	6 (13.3%)	5 (4.8%)	0.065
**Relapses in the year prior to inclusion (mean ± SD)**		0.3 ± 0.6	0.3 ± 0.6	0.3 ± 0.6	0.680
**Mean premorbid IQ score (mean ± SD)**		107.1 ± 7.3	104.4 ± 9.3	108.3 ± 5.9	0.004
**DMD therapy# (%)**	**First-line (IFN-beta,GA,DMF,TRF)**	102 (68%)	29 (64,4%)	73 (69,5%)	NS
	**Second-line (NTZ,FNG,AZA,MTX,RTX)**	23 (15,3%)	5 (11,1%)	18 (17,1%)	NS
	**Not treated**	25 (16,7%)	11 (24,4%)	14 (13,3%)	NS

EDSS: Expanded Disability Status Scale; SD: Standard Deviation; RR: relapsing-remitting; CP: chronic progressive; FSS: Fatigue Severity Scale; MADRS: Montgomery-Asberg Depression Rating Scale; IQ intelligence quotient; DMD: disease modifying drugs; IFN: interferon; GA: glatiramer acetate; DMF: dimethyl fumarate; TRF: teriflunomide; NTZ: natalizumab; FNG: fingolimod; AZA: azathioprine; MTX: methotrexate; RTX; rituximab; NS = not significant.

As for the profile of cognitive dysfunction, the domains most frequently failed by the patients were information processing speed (69 subjects, 46%), executive functioning (39, 26%), verbal learning (41, 27%) and visuo-spatial learning (26, 17%). The mean number of tests failed was 3.01 ± 2.19, the median number 2.

Tables 2 and 3 show the results of the univariate analysis regarding exposure to the hypothesized risk factors. As regard physical activity, we used the IPAQ score to categorize the subjects in 3 classes (low, moderate and high physical activity). Considering physical activity in childhood and adolescence, two (1,3%) patients didn’t practice any physical activity, while 126 (84%) practiced low level of physical activity, 15 (10%) did moderate and 7 (4,7%) high intensity physical exercise. For the statistical analysis, childhood and adolescence physical activity was considered as a dichotomized value (yes/no), since the vast majority of the patients (94%, n = 141) did low-moderate physical activity.

**Table 2:** Cardiovascular risks factors and comorbidities.

	Cognitively impaired (#45, 30%)	Cognitively preserved (#105, 70%)	*p*
**Diabetes** **# (%)**	4 (8.9%)	4 (3.8%)	0.205
**Hypertension** **# (%)**	6 (13.3%)	15 (14.3%)	0.878
**Hypercholesterolemia** **# (%)**	5 (11.1%)	11 (10.5%)	0.908
**Hypertriglyceridemia** **# (%)**	0 (0%)	8 (7.6%)	0.057
**Thyroid disease** **# (%)**	6 (13.3%)	8 (7.6%)	0.270
**Mononucleosis** **# (%)**	7 (16.7%)	26 (28.9%)	0.131
**Family History of MS** **# (%)**	9 (20%)	23 (21.9%)	0.794
**Family History of Psychiatric disorder** **# (%)**	3 (6.7%)	11 (10.5%)	0.462
**Family History of Cognitive impairment** **# (%)**	6 (13.3%)	19 (18.1%)	0.473
**BMI** **mean** ± **SD**	24.4 ± 4.9	25.0 ± 4.7	0.490
**History of brain trauma # (%)**	9 (20%)	13 (12.4%)	0.227

Hypertension was defined as blood pressure values persistently above 140/90 mmHg in different measurements, as defined in the 2018 European Society of Cardiology Guidelines [21] BMI: Body Mass Index; SD: standard deviation.

**Table 3:** Diet and lifestyle.

	Cognitively impaired (#45, 30%)	Cognitively preserved (#105, 70%)	*p*
**Diet: vegetarian/gluten free/lactose free** **# (%)**	7 (15.6%)	14 (13.3%)	0.719
**Vitamin D supplementation** **# (%)**	20 (44.4%)	52 (49.5%)	0.568
Estroprogestinic therapies* # (%)	3 (8.8%)	18 (26.1%)	0.041
**Caffeine intake** **(mean # of coffee per day** ± **SD)**	2.2 ± 1.5	2.5 ± 1.6	0.296
**Current smoking** **# (%)**	16 (35.6%)	39 (37.1%)	0.853
**Alcohol** **(mean number of drinks per day** ± **SD)**	0.5 ± 0.6	0.5 ± 0.7	0.818
**Cannabis** **# (%)**	1 (2.2%)	4 (3.8%)	0.620
**Currently physical activity** **# (%)**	27 (60%)	48 (45.7%)	0.109
**Physical activity in childhood-adolescence** **# (%)**	20 (44.4%)	71 (67.6%)	0.008
**Leisure activities** **(mean score** ± **SD)**	11.9 ± 3.9	12.0 ± 3.2	0.797

SD: standard deviation.

* Calculated on 103 women.

CI was related with lower physical activity in childhood and adolescence (44%, p = 0.008). Analyzing separately the different IPAQ categories, there was no statistical difference (data not shown).

In female patients, hormonal therapy resulted to be protective against CI (26,1%, p = 0.041).

Moreover, a multivariable analysis including only those variables that were significant in the univariate assessment (p ≤ 0.05) was conducted, and its results reported in Table 4.

**Table 4:** Multivariable logistic and linear regression model.

	OR	95% CI	*p*
**Age, years**	1.06	1.02–1.10	0.004
**Premorbid IQ**	0.93	0.88–0.98	0.004

CI: cognitive impairment. EDSS: Expanded Disability Status Scale. IQ: intelligence quotient. OR: Odds Ratio. ***Covariates in the models*:** *age*, *age at onset*, *EDSS median*, *mean premorbid IQ score*, *physical activity in childhood-adolescence*, *disease course*, *sex*.

In the multivariable analysis, the only significant variable associated with CI were older age (p = 0.004; OR 1.06; 95% CI: 1.02–1.10) and premorbid IQ (p = 0.004; OR 0.93; 95% CI: 0.88–0.98).

For female patients (n = 103), the multivariable analysis was carried out including in the model also hormonal therapy and confirmed the significant role of age (p = 0.012; OR = 1,06; 95% IC: 1.01–1.12) and premorbid IQ (p = 0.003; OR = 0.89; 95% IC: 0.83–0.96).

Removing the IQ from the model, CI was associated with higher EDSS (p = 0.030; OR = 1.25; 95% IC: 1.02–1.53), older age at onset of MS (p = 0.045; OR = 1.04; 95% IC: 1.00–1.08) and, as a trend, physical activity in childhood and adolescence (p = 0.066; OR = 0.49; 95% IC: 0.23–1.05) (Table 5).

**Table 5:** Multivariable logistic and linear regression model without IQ.

	OR	95% CI	*p*
**Age at onset, years**	1.04	1.00–1.08	0.045
**EDSS**	1.25	1.02–1.53	0.030
**Physical activity in childhood-adolescence**	0.49	0.23–1.05	0.066

CI: cognitive impairment. EDSS: Expanded Disability Status Scale. OR: Odds Ratio.

***Covariates in the models:** age, age at onset, EDSS median, physical activity in childhood-adolescence, disease course, sex*.

Some factors were analyzed in greater detail. Due to incomplete information, smoking was considered as a dichotomous variable (YES/NO) instead of number of cigarettes per day/packs for year, while, for alcohol consumption the mean number of drinks per day was used. Moreover, as for the BMI, the mean value was 24.4 ± 4.90 in cognitively impaired and 25.0 ± 4.70 in preserved patients. A BMI>25 (indicating overweight) was observed in 16 cognitively impaired (35.5%) and 35 preserved patients (33.3%); a BMI between 18 and 25 (indicating normal weight) was observed in 18 (40%) cognitively impaired and 49 (46.7%) preserved patients; finally, a BMI<18 (indicating underweight) was found respectively in 1 (2.2%) and 4 (3.8%) patients in the two groups. All the above differences were not significant.

While in the univariate analysis previous physical activity was a significant protective factor and in the multivariable analysis it showed a trend towards significance, current physical activity was not associated with CI.

Caffeine intake measured as mean number of coffees per day was comparable between impaired (2.2 ± 1.5) and preserved (2.5 ± 1.6) patients. One patient in the impaired and four patients in the preserved group reported use of cannabis, which did not reach the level of statistical significance.

Finally, considering that other studies have used two-tests failure as a cut-off to identify CI, we carried out a second multivariable analysis using this criterion to define CI. In this analysis the role of older age (p = 0.003; OR 1.06; 95% CI: 1.02–1.10) and premorbid IQ (p = 0.011; OR 0.93; 95% CI: 0.88–0.98) was confirmed together with the association of CI with thyroid diseases (p = 0.040; OR 4.52; 95% CI: 1.07–19.09). These were represented by eight cases of hypothyroidism and one case of hyperthyroidism. In these patients, the actual hormonal levels at the time of the study were however in the normal range.

## Discussion

There is limited information about risk factors or protective factors associated with CI in MS. Among demographic and clinical correlates, aging is clearly associated to a decline of neuropsychological performance [22],while conflicting evidence suggests that male sex is a risk factor for progression of CI [23]. Fatigue, depression and disease duration are weakly correlated with cognitive capacity [24]. CI is more frequent in patients who are in the progressive phase of the disease and the profile and severity of cognitive deficits seem to be mostly driven by age and disability accrual [22]. Several neuroimaging studies have extensively explored MRI correlates of CI in MS, highlighting the relevance of white and grey matter changes and, in particular, brain volume loss [25].

Our results are in line with previous cross-sectional and longitudinal observations pointing to the association of CI with older age at onset, aging and disability [22,26]. Early and appropriate treatment of the disease with disease modifying drugs can therefore represent a key strategy to improve both the physical and cognitive outcome of the subject [27].

As for comorbidities, they have been associated with a worse disease outcome [28]. In particular, cardiovascular risk factors have been associated with brain lesion burden and brain atrophy [29]. In our sample, using the failure of two tests as the cut-off point to define CI, a history of thyroid disease was associated with poorer cognitive performance. While further studies should better analyze this association, thyroid dysfunction represents a relevant, modifiable risk factor that is common in young adults with MS.

Among lifestyle factors, smoking is a well-recognized risk factor for Alzheimer disease and is related to preclinical changes in the brain, higher risk of cognitive decline, and increased risk of dementia [30]. In MS smoking is both a susceptibility risk factor for MS and a prognostic factor, associated with disease progression. Moreover, it has been associated with increased lesion volumes and brain atrophy in multiple sclerosis [31] and CI in one study [32]. Prolonged use of inhaled or ingested street cannabis in patients with MS has been associated with poor performance on cognitive domains commonly affected in this population [33]. In a neuropsychological and f-MRI study [34] cannabis use was associated with compromised cerebral compensatory mechanisms, already faulty in MS. Lastly, regarding alcohol consumption, chronic heavy intake is a well-established cause of brain atrophy and dementia [35] although this association has not been specifically explored in MS.

In our study smoking, cannabis and alcohol intake were not associated with cognitive functioning. In our sample, however, heavy drinkers were not represented. Moreover, we can hypothesize that, on the one hand, our negative results may be due to relatively small sample size and, to the other hand, that diagnosis of a chronic disease itself may have led the patient to modify his/her lifestyle orienting the subject towards healthier inhabits. Finally, we cannot rule out the possibility of under-report in our patients, particularly for alcohol or cannabis use.

As for potential protective factors, in our study higher cognitive reserve stands out as the most consistent, potentially modifiable protective factor. In fact, in all the analyses, better cognitive performance was associated with higher cognitive reserve—expressed as premorbid IQ—which confirms the findings of other studies [36,37]. In a previous work [37] we found that the protective role of cognitive reserve mainly applied to the early stages of the disease and within a hypothetical “threshold” of brain atrophy. However, in our sample of subjects with a mean age of 45 years and a mean disease duration of 11 years this protective role was still evident, highlighting the potential of preventive strategies focusing on intellectual enrichment in this population of patients.

It is noteworthy that in our sample hormonal therapy in female patients resulted to be protective against CI. This was represented by estroprogestinic oral contraceptives whose exact dosage and duration of intake were, however, not recorded. A few randomized clinical trials have reported positive effects of estrogen therapy on cognitive performance [38–42] and the topic has been extensively reviewed elsewhere [43,44]. In animal studies the estrogens has been showed to have task-specific effects on cognitive performance and these effects might be influenced by age and time after loss of ovarian function [45]. One of the proposed mechanisms is that estradiol may enhance performance by increasing cholinergic activity in the hippocampus and cerebral cortex. Furthermore, estradiol effects on hippocampal neurons might be modulated by cholinergic activity, enabling this hormone to produce lasting changes in cortical connectivity and function [45]. These effects might be moderated by aging, that decreases cholinergic activity [45].

Hormonal therapy was not retained in our multivariable analysis, and that—in light of the younger age of the women taking estroprogestinics in our sample—might be due to the more important influence of aging, rather than the hormonal treatment, on cognitive functions.

In our sample, it is intriguing that physical activity in childhood and adolescence emerged as a protective factor in the univariate and remained as a trend in the multivariable analysis. However, in our study we could not document the potential impact of different levels of physical activity, possibly due to the relatively small sample size and the low proportion of patients engaged in moderate or high intensity activity.

There is growing evidence deriving from animal studies [46] and human studies in children and elderly people [47] that physical exercise is connected with greater hippocampal volume, higher white matter integrity and more efficient white matter activity [47]. Thus, physical activity may improve cognitive functioning and memory, possibly enhancing hippocampal function [48]. In MS, physical exercise may be protective against the development of cognitive dysfunction and exert a synergistic effect together with cognitive rehabilitation in patients with established CI [49]. However, there is limited information on the potential role of physical activity in childhood and adolescence. In the general population this has been associated with reduced morbidity [50], including a decreased risk of MS in a recent study [51]. In the context of cognitive functioning, physical exercise early in life might contribute to the building of the subject cognitive reserve. On the other hand, physical activity could be related to higher socio-economic status, an acknowledged proxy of cognitive reserve. Based on our findings, further studies focusing on the pediatric age, using a precise classification of physical exercise in terms of intensity and duration appear to be highly advisable.

Furthermore, we could not demonstrate any protective role of caffeine intake, that in healthy adults has been associated with improvement of alertness, vigilance, attention and reaction time, and, less consistently, memory and higher-order executive functions [52].

Finally, we did not explore the potential impact on cognition of different levels of vitamin D, whereas one MS study has suggested an association between vitamin D supplementation and better cognitive performance [53].

Our study has a few limitations, mainly represented by the cross-sectional design and retrospective assessment of exposures and potential recall bias. Therefore, the results need to be confirmed in a larger sample with a more accurate assessment of exposure to hypothesized factors. Finally, the statistical method used—the stepwise multivariable logistic analysis–although widely used and appealing because of its simplicity may have some limitations. In fact, it may be less efficient especially for small studies, where problems such as over-fitting due to data sparsity and collinearity may arise. Another caveat is the potential bias due to confounding [54] Causal diagrams utilize assumptions regarding the underlying causal relationships between relevant variables to perform confounder selection rather than relying on observed statistical associations. In this respect, the use of multiple models based on multiple potential direct acyclic graphs (DAGs) could be preferred [55]

Since MS is a multifactorial disease deriving from a complex interplay between genetic and environmental factors, further investigation is also required on potential genetic susceptibility to CI [56].

In conclusion, this exploratory study underscores the complexity of physiopathological mechanisms underlying CI in MS and provides a few clues to further research in this area.

## Supporting information

S1 File Raw data. Fully anonymized dataset. (SAV)

S2 File Semi-structured interview. The interview used for the study purposes.
(DOC)
