# Correction: Cognitive impairment in multiple sclerosis: An exploratory analysis of environmental and lifestyle risk factors

**DOI:** 10.1371/journal.pone.0225494

**Published:** 2019-11-14

**Authors:** Maria Pia Amato, Elio Prestipino, Angelo Bellinvia, Claudia Niccolai, Lorenzo Razzolini, Luisa Pastò, Roberto Fratangelo, Laura Tudisco, Mattia Fonderico, Paolo Luca Mattiolo, Benedetta Goretti, Giovanni Bosco Zimatore, Nunzia Alessandra Losignore, Emilio Portaccio, Francesco Lolli

The affiliation for the fourth author is incorrect. Claudia Niccolai is not affiliated with #1 but with #2: IRCCS Fondazione Don Carlo Gnocchi, Florence, Italy.
