# Supplementary material for: Cognitive impairment in multiple sclerosis: An exploratory analysis of environmental and lifestyle risk factors
Source: PLoS One. 2019 Oct 21;14(10):e0222929. doi: 10.1371/journal.pone.0222929 (PMC6802833; doi:10.1371/journal.pone.0222929)
Supplement: S2 File — The interview used for the study purposes. (DOC) [file pone.0222929.s002.doc]

**Demographic data**

Name ________________________________________________

Surname _____________________________________________

Date of birth _________________________________________

Telephone number ______________________________________________

Date of cognitive evaluation ________________________________

Ethnicity ________________________________________________

Marital status ____________________________________________

Cohabitants ________________________________________

**Cardiovascular risk factors**

Weight (Kg) ______

Height (cm) ______

BMI _______

Diabetes mellitus NO YES

If yes, specify disease duration and medications

____________________________________________

____________________________________________

____________________________________________

Arterial hypertension NO YES

If yes, specify disease duration and medications

____________________________________________

____________________________________________

____________________________________________

Hypercholesterolemia NO YES

If yes, specify disease duration and medications

____________________________________________

____________________________________________

____________________________________________

Hypertriglyceridemia NO YES

If yes, specify disease duration and medications

____________________________________________

____________________________________________

____________________________________________

Smoking (last year) NO YES

If yes, specify number of cigarettes smoked per day

____________________________________________

____________________________________________

____________________________________________

Smoking (more than one year ago)

NO YES

If yes, specify number of cigarettes/day and years of
 smoking

____________________________________________

____________________________________________

____________________________________________

**Family history**

Cardiovascular events in young age (males <55y, females < 65y)

NO YES

If yes, specify, disease the degree of kinship

____________________________________________

____________________________________________

____________________________________________

Multiple sclerosis NO YES

If yes, specify the degree of kinship

____________________________________________

____________________________________________

____________________________________________

Psychiatric disorders NO YES

If yes, specify diagnosis and degree of kinship

____________________________________________

____________________________________________

____________________________________________

Cognitive impairment/Dementia NO YES

If yes, specify diagnosis and degree of kinship

____________________________________________

____________________________________________

____________________________________________

Years of education Mother ____________________________________________

Father _____________________________________________

Level of education Mother ____________________________________________

Father _____________________________________________

Working activity (specify previous and actual occupation and if part-time or full-time employment)

Mother ____________________________________________

__________________________________________________

__________________________________________________

Father _____________________________________________

__________________________________________________

__________________________________________________

Cohabitants’ years of education, educational level and working activity (previous and actual occupation, part-time or full-time) __________________________________________________________________________________________________________________________________________________________________________________________________________________________________________________________________________________________________________

**Past medical history and comorbidities**

Infectious mononucleosis NO YES

If yes specify age at onset __________________________________________________

__________________________________________________

__________________________________________________

Head trauma NO YES

If yes specify age, complications and neurolopsychological sequelae __________________________________________________________________________________________________________________________________________________________________________________________________________________________________________________________

Hepatitis NO YES

If yes specify type, age at onset and medications

__________________________________________________

__________________________________________________

__________________________________________________

Thyroid disorders NO YES

If yes specify age at diagnosis, diagnosis and medications __________________________________________________

__________________________________________________

__________________________________________________

Psychiatric disorders NO YES

If yes specify age, diagnosis and medications __________________________________________________

__________________________________________________

__________________________________________________

Autoimmune comorbidities NO YES

If yes specify age, diagnosis and medications

__________________________________________________

__________________________________________________

__________________________________________________

Other comorbidities NO YES

If yes specify age, diagnosis and medications

__________________________________________________

__________________________________________________

__________________________________________________

**Schooling, work, *leisure activities***

Years of Education__________________________________________________________

Educational qualification____________________________________________________________

Past physical activity NO YES

If yes, specify

Past physical activity (age of onset, type, session length and frequency, years of duration)

________________________________________________________________________________________________________________________________________________________________________________________________________________________________________________________________________________________________________________________________________________________________________________________________________________

Current physical activity NO YES

If yes, specify

Current physical activity (age of onset, type, session length, weekly frequency, years of duration)______________________________________________________________________________________________________________________________________________________________________________________________________________________________________________________________________________________________________________________

Working Activities (Specify previous and actual occupation and if full time or part time) ________________________________________________________________________________________________________________________________________________________________________________________________________________________________________________________________________________________________________________________________________________________________________________________________________________

Leisure Activities (Sumowski et al, 2010)

|  | 1 | 2 | 3 | 4 | 5 |
| --- | --- | --- | --- | --- | --- |
| Book reading |  |  |  |  |  |
| Newspaper and magazines reading |  |  |  |  |  |
| Artistic production^1^ |  |  |  |  |  |
| Writing^2^ |  |  |  |  |  |
| Play an instrument |  |  |  |  |  |
| Structured Games^3^ |  |  |  |  |  |
| Hobbies^4^ |  |  |  |  |  |

Once or less yearly 1

Sometimes yearly 2

Sometimes monthly 3

Sometimes weekly 4

Daily 5

TOTAL = ________

^1^Ex.: painting, poetry, sculpture, composing songs, ballet, etc.

^2^Ex.: diary, blogs, newsletters, reports, essays, etc.

^3^Ex.: chess, cards, puzzles, board games

^4^Ex.: gardening, collecting, modeling

**Diet and Therapy**

Special Diet NO YES

If yes, specify type of diet, age at onset

__________________________________________________ __________________________________________________ __________________________________________________

Alcohol intake in the last year NO YES

If yes, specify type, frequency, how many drinks each time

__________________________________________________ __________________________________________________ __________________________________________________

Alcohol intake (more

than one year ago) NO YES

If yes, specify type, frequency, how many drinks each time, duration (years)

__________________________________________________ __________________________________________________ __________________________________________________

Coffee Intake NO YES

If yes, how many cups of coffee daily

__________________________________________________ __________________________________________________ __________________________________________________

Illicit drugs intake NO YES

If yes, specify type and frequency, age at onset, duration (years)

__________________________________________________ __________________________________________________ __________________________________________________ __________________________________________________

Current Therapy

________________________________________________________________________________________________________________________________________________________________________________________________________________________________________________________________________________________________________________________________________________________________________________________________________________

Hormone/Estroprogestinic Therapy (current or previous, specify type and duration)

________________________________________________________________________________________________________________________________________________________________________________________________________________________________________________________________________________________________________________________________________________________________________________________________________________

Current vitamine D supplementation (specify dosage, frequency and duration)

________________________________________________________________________________________________________________________________________________________________________________________________________________________________________________________________________________________________________________________________
